# Outcomes and Management of Positive Margins in Chondrosarcoma With Soft Tissue Extension: A Case Series and Review of Literature

**DOI:** 10.1002/cnr2.70148

**Published:** 2025-03-11

**Authors:** Austin Yu, Trevor Poulson, Zachary Butler, Matthew Demetrious, Steven Gitelis, Alan T. Blank

**Affiliations:** ^1^ Department of Orthopedic Surgery, Section of Orthopedic Oncology Rush University Medical Center Chicago Illinois USA; ^2^ Department of Pathology Rush University Medical Center Chicago Illinois USA

**Keywords:** chondrosarcoma, extraosseous extension, positive margins, soft tissue extension

## Abstract

**Background:**

Chondrosarcoma accounts for 20% of all bone sarcomas and may present with soft tissue extension. The presence of an extraosseous component, along with positive surgical margins, independently have been associated with increased risk of local recurrence and decreased survival. The purpose of this investigation is to describe the treatment and outcomes of six chondrosarcoma patients who presented with chondrosarcoma with soft tissue extension along with positive surgical margins post negative en bloc resection.

**Case:**

This was a retrospective review over a consecutive 13‐year period. Data including treatment details and outcomes were included. All patients underwent attempted negative margin en bloc resection and encountered unplanned positive margins on intraoperative determination or postoperative pathology (R1). A total of six cases were identified. Average age (SD) was 61.8 years (6.11) with median (IQR) follow‐up of 17.0 months (10.3–39.5). Three (50.0%) cases arose in the extremities, and 3 (50.0%) cases in the pelvis. All patients underwent attempted negative margin en bloc resection. Three (50.0%) cases recurred with median (IQR) time to recurrence of 10.0 months (9.0–31.0). At study conclusion, 5 (83.3%) were alive with median (IQR) survival of 20.5 months (11.3–41.0).

**Conclusion:**

Despite limited sample size, our data reflected a significantly higher recurrence rate compared to either chondrosarcomas with positive margins or extraosseous extension. Our cohort represents a high‐risk subgroup of chondrosarcoma patients, which may dictate increased monitoring and guide future treatment recommendations for these patients.

## 1 Introduction

Chondrosarcomas are malignant tumors derived from cartilage, accounting for 20% of all bony sarcomas. Subtypes of chondrosarcoma include conventional, clear cell, mesenchymal, or dedifferentiated types. Often they present with significant pain and present with notable radiographic findings such as thickening of the bone cortex, cortical destruction, and extraosseous extension (EOE) into the surrounding soft tissue [1, 2, 3]. Negative margin surgical excision is the primary treatment option for chondrosarcoma in order to prevent their recurrence and spread.

Ideally, surgery is performed in the attempt to create negative or “clear” margins, in which sufficient tissue is resected, leaving the surrounding tissue histologically proven to be clear of tumor [4]. However, in certain cases positive margins may remain. This may be due to a lack of correct diagnosis, patient preference, or a difficult anatomic area, resulting in an unplanned positive margin [4]. In such cases, inadequate margins significantly increase the risk of recurrence as well as patient mortality with estimated 5‐year survival of 63% [5, 6, 7, 8, 9].

While obtaining negative margins of resection has proven to be critical for patient survival and recurrence, there have been few studies focused on the survivorship and recurrence rate of chondrosarcomas with EOE [10, 11]. Studies have previously reported EOE in chondrosarcoma to be a significant risk factor on overall survival (OS), metastasis, and local recurrence [12, 13, 14]. However, no study has reported specific recurrence or OS rates on those with the combination of EOE and positive surgical margins.

Given the limited clinical outcomes data of this patient subset, the primary goal is to calculate the recurrence rate of chondrosarcoma patients with the combination of EOE and positive margins after resection, while also establishing the scientific literature on this subset, which is known to have worse outcomes but has been underrepresented in research. In this case series, we describe our institution's experience in patient management as well as a review of the current literature. We report higher incidence of recurrence than chondrosarcoma with positive margins alone.

## 2 Methods

Following Institutional Review Board approval, a musculoskeletal pathologist used natural language search, a search tool that allows retrieval of patients given a diagnosis prompt, to identify all cases of patients with chondrosarcoma over the age of 18 years within our pathology reports from 2010 to 2023. A total of 192 patients were retrieved. All patients with benign chondromas, non‐cartilaginous tumors, equivocal histopathological diagnosis, or incomplete medical records were excluded, resulting in 112 patients. Electronic medical records of the remaining patients were reviewed for the presence of positive surgical margins and EOE. Fifty‐eight (51.8%) patients presented with neither characteristic, 41 (36.6%) presented with positive margins only, 7 (6.3%) presented with EOE only, and 6 (6.3%) presented with both characteristics, which were included in this study. Confirmation of margins and EOE was made through careful review of the patient's imaging, histopathology report—R1 positive margins, and clinical note.

### 2.1 Statistical Analyses

Demographic and clinicopathologic data were analyzed using descriptive statistics. Continuous variables of interest were represented as the mean, standard deviation (SD), or median, range. Survival was recorded from the time of biopsy until date of death or last known follow‐up without death having occurred. Recurrence time was calculated from the time of surgical intervention until date of local or systemic relapse (event). Due to the limited size of our cohort, we were unable to perform a meaningful statistically powered analysis.

## 3 Results

Pooled patient characteristics are listed in Table 1, and individual patient characteristics, treatments, and treatment outcomes are listed in Table 2. Mean age and standard deviation (SD) was 61.8 (6.11) years. All six cases initially underwent attempted negative en bloc resection. Fifty percent (*n* = 3) of tumors were high‐grade and 50.0% (*n* = 3) were intermediate grade. Thirty‐three percent (*n* = 2) of tumors occurred in the femur, 50.0% (*n* = 3) in the pelvis, and 16.7% (*n* = 1) in the humerus. Average tumor size, measured by greatest dimension, was 9.45 cm (SD 5.86). Soft tissue extension with positive margins after resection, confirmed with histopathology (R1), was found in all six patients after their index procedure. Three patients had positive margins at the soft tissue margin, while the other three were at the osseous margin. Eighty‐three percent (*n* = 5) of people received additional treatment. One patient received adjuvant chemotherapy alone, two patients received adjuvant radiotherapy alone, and two patients received both. For adjuvant chemotherapy, all patients (*n* = 3) received cisplatin and doxorubicin (AP). The one patient that did not receive additional treatment underwent a hemipelvectomy with ischial margins positive for tumor and tumor extension through the soft tissue. This patient was given the option for re‐excision or observation and opted for observation.

**TABLE 1:** Patient demographics.

Age^a^	61.8	6.11
Race		
White	4	66.6%
Black	1	16.7%
Other	1	16.7%
Sex		
Male	2	33.3%
Female	4	66.7%
Location		
Femur	2	33.3%
Pelvis	3	50.0%
Humerus	1	16.7%
Initial biopsy	6	100.0%
Size^a^	9.45	5.86
Grade		
1	0	0.0%
2	3	50.0%
3	3	50.0%
Dedifferentiation		
Yes	3	50.0%
No	3	50.0%
Adjuvant radiation	2	33.3%
Adjuvant chemotherapy	3	50.0%
Recurrence	3	50.0%
Time to recurrence^b^	10	(9.0–31.0)
Metastasis	3	50.0%
Time to metastasis^b^	14	(7.5–21.0)
Mortality	1	16.7%
Follow‐up (months)^b^	17	(10.3–39.5)
Survival (months)^b^	20.5	(11.3–41.0)

^a^ Mean, standard deviation.

^b^ Median, interquartile range.

**TABLE 2:** Patient characteristics.

Age	Sex	Grade	1° tumor location	Metastatic at presentation	Chemo	RT	Recurrence	Dedifferentiation	Metastasis	Follow‐up (months)	Survival (months)	Status
64	F	3	Pelvis	No	Adj	Adj	Yes	Yes	No	17	23	Alive
60	F	3	Humerus	No	Adj	No	No	Yes (on presentation)	Yes	8	9	Alive
51	M	3	Femur	No	No	Adj	Yes	Yes	Yes	91	91	Alive
62	M	2	Femur	No	No	Adj	No	No	No	6	7	Alive
65	F	2	Pelvis	No	Adj	Adj	Yes	No	Yes	17	18	Dead
69	F	2	Pelvis	No	No	No	No	No	No	47	47	Alive

Local recurrence occurred in 50.0% (*n* = 3) patients with a median time and interquartile range (IQR) to recurrence of 10 months (9.0–31.0). Three experienced metastasis either to the lungs (*n* = 2) or ribs and paraspinal region (*n* = 1) with median time to metastasis (IQR) of 14.0 months (7.5–21.0). Patients with lung metastasis received video‐assisted thoracic surgery (VATS) lung wedge resection. The patient with rib and paraspinal metastasis was discovered after imaging noted a large lobular mass in the left 4th rib notable for bone marrow involvement with soft tissue extension. The patient was treated with an extended posterolateral thoracotomy with negative en bloc resection of ribs 3, 4, and 5, however, paraspinal positive margins were noted. Metastasis subsequently spread to the lumbar and thoracic spinal regions as well as the occipital bone. Dedifferentiation was noted in four patients. One patient was found with high‐grade dedifferentiated chondrosarcoma on presentation, and the other three were noted later with median time to differentiation of 8 months.

### 3.1 Outcomes

Median follow‐up (IQR) was 17 months (10.3–39.5). At the conclusion of this study, 16.7% (*n* = 1) patients were deceased, 66.6% (*n* = 4) patients were alive with active disease, and 16.7% (*n* = 1) patient were alive but did not pursue further treatment.

## 4 Discussion

In our literature review we examined 18 studies analyzing the impact of positive margins or EOE on patient survival and recurrence. Positive margins were not uniformly defined across the literature. Two studies [9, 13] reported margins using the residual tumor (R) classification system defined by the Union for International Cancer Control (UICC) [15]. R0 is defined as residual tumor, R1 microscopic residual tumor, and R2 macroscopic tumor. A R0 margin has been described as a strong predictor of a favorable prognosis [16], and both studies defined an R1/R2 margin as containing a positive margin. Tsuda et al. categorized margin distance as 0 mm, 0.1–4 mm, and > 4 mm, noting a significant impact on sarcoma‐specific death and disease progression with 0 mm margins. Four studies defined positive margins as intralesional excision or obtaining marginal margins during resection [8, 17, 18, 19]. Matheron et al. explicitly focused on inadvertent positive margins (IPMs), also known as unplanned positive margins, which describes a sarcoma planned for complete resection but returned positive margins on histopathology. IPMs have described as a category in the Toronto Margin Context Classification (TMCC) system both for bony tumors as well as soft tissue tumors [4, 20, 21]. We defined presence of a positive margin based upon careful inspection of clinical imaging and histopathological analysis postoperatively.

EOE occurs in up to 50% high‐grade chondrosarcomas, which may present with cortical bone destruction and a moth‐eaten appearance on radiographs [2]. The presence of an EOE was described in six studies, described as presence of a soft tissue extension [12, 22, 23], extracompartmental extension [24], or extraosseous component with cortical breach [14]. Studies primarily focused on either central conventional chondrosarcoma or high‐grade dedifferentiated chondrosarcoma. Two studies further included peripheral chondrosarcoma, described by Wellings et al. as a secondary tumor arising from a previous osteochondroma or enchondroma [12, 23]. In our cohort three patients were categorized as conventional chondrosarcomas, one patient presented with dedifferentiated chondrosarcoma, and three patients presented with conventional chondrosarcoma with subsequent dedifferentiation during the study period.

The presence of positive margins has widely been cited as a significant risk factor for OS and recurrence across multiple chondrosarcoma types [2, 5, 7, 11, 13, 25, 26, 27]. Therefore, obtaining negative resection margins is key to favorable long‐term prognosis. Alvarado et al. found large tumor size significantly impacted the likelihood of positive surgical margins in chest wall chondrosarcomas [5]. They hypothesized this was most likely due to increased tumor invasion into surrounding structures, increasing the difficulty in achieving complete resection. Similarly, pelvic chondrosarcomas present a higher incidence of positive margins due to the complex anatomy of surrounding neurovascular structures [2]. To achieve adequate margins, limb‐salvage options such as internal hemipelvectomy or hind‐quarter amputation are recommended [2].

EOE was also established as significantly impacting OS [12, 13, 14, 23, 24] and recurrence [12, 14]. Fiorenza et al. theorized this impact on survival could be associated with the inherent severity of high‐grade tumors, which often present with EOE. However, EOE remained significant after multivariate analysis and demonstrated a worse prognosis compared to intracompartmental‐only high‐grade chondrosarcoma. Similarly, a prospective sarcoma database study found that 40.8% of patients with pure intraosseous chondrosarcomas were grade two or three, compared to 85% of those with chondrosarcomas involving EOE [14]. These findings support EOE as an independent risk factor to tumor prognosis. Our patient cohort presented with both EOE and IPMs, representing a high‐risk population that has not previously been examined to our knowledge.

In chondrosarcoma with positive margins, while recurrence rates ranged from 30.4%–75% [4, 8, 19, 24, 26, 27]. In patients with EOE, recurrence data was limited. Fiorenza et al. noted a recurrence rate of 46%, In our cohort, we reported an overall cohort recurrence rate of 50.0% (3/6). Despite a limited sample size, this data suggest the addition of EOE may significantly increase the chance of recurrence. While this is not a surprising finding, there is very limited published data on this subset of patients, emphasizing the importance of contributing to the existing body of knowledge.

Additional prognostic factors may be viewed in Table 3. Tumor grade, including dedifferentiated chondrosarcoma, was cited as significantly increasing overall mortality, local recurrence, and metastasis [18, 22, 24, 25, 26]. Regarding tumor location, the proximal femur was cited as a significant risk factor for increased local recurrence rates [9, 14]. Laitinen ascribed this due to difficulty in achieving complete resection near the greater trochanter, while Nakayama et al. reported high rates of misdiagnosis in the femoral head, leading to inadequate marginal resection and subsequent local recurrence. One patient in our cohort was diagnosed in chondrosarcoma in the proximal femur and underwent resection with a proximal femoral reconstruction (PFR). From this procedure, the patient did not experience local recurrence or metastasis, demonstrating positive outcomes with complete endoprosthetic reconstruction.

**TABLE 3:** Survival and recurrence of positive margins or EOE according to the literature.

Author	Positive margins or extraosseous extension	Year	How positive margin/extraosseous extension was defined	*n* total	*n* with positive margins or extraosseous extension	Survival (include hazard ratio if no %)	Recurrence (same)	Chondrosarcoma type; location	Additional prognostic factors
Laitinen et al. [14]	Extraosseous extension	2024	Extraosseous component with cortical breach	Grade 1: 57	1	10‐year DSS: 100%	1‐year LRFS: 100% 5‐year LRFS: 88% 10‐year LRFS: 88%	Central; femur	The location of the extraosseous component within the femur significantly affected local recurrence rates. Adequate margins were especially hard to achieve around the greater trochanter. Decrease LRFS: histological grade, narrow surgical margin (< 3 mm). DSS: histological grade, LR, metastases, surgical margin
				Grade 2: 94	17	1‐year DSS: 100% 5‐year DSS: 78% 10‐year DSS: 76%	1‐year LRFS: 95% 5‐year LRFS: 70% 10‐year LRFS: 70%	Central; femur	
				Grade 3: 51	10	1‐year DSS: 92% 5‐year DSS: 68% 10‐year DSS: 50%	1‐year LRFS: 82% 5‐year LRFS: 69% 10‐year LRFS: 69%	Central; femur	
Wellings et al. [23]	Extraosseous extension	2021	Presence of soft tissue mass	39	26	All patient deaths had a soft tissue component	LR HR 1.72 (95% CI 0.33–9.01)	Grade 1, 2, 3, dedifferentiated, central, peripheral; scapula	High grade associated with mortality, local recurrence associated with metastasis
	Positive margins		Not specified		5	DSS HR 0.85 (95% CI 0.10–6.99)	LR HR 8.85 (95% CI 1.97–39.69)		
Aggerholm‐Pedersen et al. [22]	Extraosseous extension	2019	Presence of soft tissue extension	199	148	Overall mortality HR: 1.5 (95% CI 0.6–3.8); DSS HR: 1.2 (95% CI 0.4–3.7)	—	Conventional, dedifferentiated	Presence of comorbidity, high‐grade tumor associated with significantly increased overall mortality and disease‐specific mortality
Thorkildsen et al. [12]	Extraosseous extension	2019	Presence of soft tissue component	311	10	DSS HR 2.82 (1.09–7.28)	LR HR 3.20 (1.07–9.57)	Central, peripheral, dedifferentiated, periosteal; head & neck	Presence of soft tissue component predictor of metastasis as well
Miao et al. [13]	Extraosseous extension	2019	Not specified	72	69	OS 12.8 months	PFS 6.4 months	Dedifferentiated	Chemotherapy with doxorubicin and cisplatin improved PFS
	Positive margins		R1/R2 margins		13	OS 12.3 months	PFS 6.6 months		
Fiorenza et al. [24]	Extraosseous extension	2002	Extracompartmental component	194	118	5‐year OS 72%	LR rate 29%	Conventional; long bones, pelvis	High‐grade, size > 10 cm, and presence of local recurrence had significant impact on overall survival, local recurrence, and metastases
	Positive margins		Inadequate margins (intralesional/marginal/undetermined)		83	5‐year OS 77%	LR rate 46%		
Alvarado et al. [5]	Positive margins	2022	Microscopic or macroscopic residual tumor	1934	159	5‐year OS 62.5%; HR 2.34 (1.73–3.18)	—	Periosteal, myxoid, mesenchymal, clear cell, dedifferentiated; chest wall	Large tumor size associated with increased risk of positive margins (OR 1.05, CI 1.011–1.075)
Evans et al. [7]	Positive margins	2022	Not specified	6653	811	Mortality OR 2.75 (1.55–4.90)	—	Dedifferentiated, myxoid, clear cell, mesenchymal, peripheral	Increased age, increased AJCC (American Joint Committee on Cancer) stage, postoperative length of stay
Matheron et al. [4]	Positive margins	2022	Unplanned positive margin	23 (4 chondrosarcoma)	7 (mixed tumor type)	—	Overall recurrence rate 30.4%	Mixed bony and soft tissue sarcomas	—
Flint et al. [17]	Positive margins	2020	Intralesional/curettage or marginal	15	5	Median time to recurrence or death: 7.73 years (95% CI 4.49–NA)	—	Clear cell	Improved outcome when initially treated by experienced musculoskeletal oncologist
Nakayama et al. [9]	Positive margins	2020	R1/R2 margins	42	3	5‐year OS: 89% (95% CI 74–96); 5‐year DFS: 77% (95% CI 58–89)	5‐year LRFS: 86% (95% CI 68–95)	Clear cell	Clear cell chondrosarcoma in the femoral head presented with the highest rate of misdiagnosis as a benign lesion, leading to inadequate margins and increased local recurrence.
Tsuda et al. [11]	Positive margins	2019	0 mm, 0.1–4 mm, > 4 mm	612	61	CISSD HR: 2.47 (95% CI 1.31–4.63)	CIDP: 2.36 (95% CI 1.41–3.95)	Central, dedifferentiated	Age at diagnosis and grade were also found to have significant effects on CISSD and CIDP in multivariate analyses.
Bindiganavile et al. [18]	Positive margins	2015	Intralesional/marginal	125	53	5‐year DSS 97.8% ± 2.2% (not significant)	5‐year LRFS 84.9% ± 4.9% (not significant)	Conventional, clear cell and periosteal, dedifferentiated, mesenchymal, secondary	Dedifferentiated type demonstrated the worst outcome. Axial location and high‐grade were predictors of worse outcomes in conventional chondrosarcoma
Roos et al. [25]	Positive margins	2015	R1/R2 margins	76	13	OS HR: 2.92 (1.03–8.25)	LR HR: 2.81 (0.92–8.64)	Chondrosarcoma; rib	Worse outcomes in tumors > 5 cm and grade 3 chondrosarcoma.
Xu et al. [19]	Positive margins	2015	Intralesional surgery with positive margins	107	15	—	Overall recurrence rate 66.7% (10/15)	Mesenchymal	5/15 received postoperative radiation, 2 recurred. 10/15 did not receive postoperative radiation, 8 recurred.
Fong et al. [27]	Positive margins	2004	Inadequate margins	24	4	5‐year survival rate: 50%; RR: 15.77 (95% CI 1.267–196.283)	Recurrence rate: 75% (3/4); RR of LR: 11.433 (95% CI 1.533–85.278)	Unspecified type; chest wall	An inadequate margin of resection was associated with a significantly worse OS and a higher chance of LR.
Rizzo et al. [8]	Positive margins	2001	Marginal margins or intralesional resection	101	23	Death from disease: 30%	LR: 35%	Grade 1, 2, or 3 chondrosarcoma	Significant association between MIB‐1 expression and recurrence/death. Local recurrence, metastases, and disease‐related death was significantly higher in the group of patients with marginal or incomplete tumor excision.
Sheth et al. [26]	Positive margins	1996	If tumor was present at inked margin microscopically	67	28	RR 3.3 (95% CI 1.7–6.6)	Recurrence rate: 54% (15/28)	Grade 1, 2, 3 and dedifferentiated; pelvis	Increased risk of LR was associated with inadequate surgical margin, tumor epicenter in the pubis, and high‐grade histology. Histological grade was associated with occurrence of distant metastasis.

Abbreviations: CI, confidence interval; CIDP, cumulative incidence of disease progression; CISSD, cumulative incidence of sarcoma‐specific death; DFS, disease‐free survival; DSS, disease‐specific survival; HR, hazard ratio; LR, local recurrence; LRFS, local recurrence‐free survival; OR, odds ratio; OS, overall survival; RR, relative risk.

### 4.1 Limitations

This case series is subject to several limitations. Firstly, our study is subject to inherent biases present in retrospective studies. Because our patient cohort represented a chondrosarcoma with highly specific tumor characteristics, we were only able to obtain a limited number of patients. Patients were collected over a 13‐year period at a tertiary referral institution, highlighting the rarity of this subtype. The small sample size limited our survival analysis with large confidence intervals, which may be better elucidated with a larger cohort study. While our data reflected similar OS to chondrosarcoma with positive margins alone, future studies should compare our chondrosarcoma cohort against a control group of intermediate and high‐grade chondrosarcomas without either tumor characteristic as well as chondrosarcomas with either positive margins alone versus EOE alone. To better elucidate their role in survival outcomes, further multicentered trials may help to answer these future research questions.

## 5 Conclusion

We present a unique cohort of high‐risk patients who exhibited EOE as well as underwent surgical resection with positive margins of intermediate and high‐grade chondrosarcoma. Despite a limited sample size, our data reflected a significantly higher recurrence rate. Our cohort represents a high‐risk subgroup of chondrosarcoma patients, highlighting the importance of increased monitoring to help prevent the heightened risk of recurrence and guide future treatment recommendations for these patients.

## Author Contributions

**Austin Yu:** data curation (equal), formal analysis (equal), investigation (equal), methodology (equal), writing – original draft (equal), writing – review and editing (equal). **Trevor Poulson:** data curation (equal), investigation (equal), writing – original draft (equal), writing – review and editing (equal). **Zachary Butler:** data curation (equal), writing – original draft (equal), writing – review and editing (equal). **Matthew Demetrious:** data curation (equal), writing – original draft (equal), writing – review and editing (equal). **Steven Gitelis:** investigation (equal), methodology (equal), project administration (equal), supervision (equal), validation (equal). **Alan T. Blank:** conceptualization (equal), formal analysis (equal), investigation (equal), methodology (equal), project administration (equal), resources (equal), supervision (equal), validation (equal), visualization (equal), writing – original draft (equal), writing – review and editing (equal).

## Ethics Statement

Rush University Medical Center obtained Institutional Review Board approval prior to beginning any research efforts. This study qualifies for exemption under 45 CFR 46.104(d) (4) as determined by the IRB.

## Conflicts of Interest

The authors declare no conflicts of interest.

## Data Availability

The data that support the findings of this study are available from the corresponding author upon reasonable request.
